# Building an R&D chemical registration system

**DOI:** 10.1186/1758-2946-4-11

**Published:** 2012-05-31

**Authors:** Elyette Martin, Aurélien Monge, Jacques-Antoine Duret, Federico Gualandi, Manuel C Peitsch, Pavel Pospisil

**Affiliations:** 1 Philip Morris International R&D, Philip Morris Products S.A, Neuchâtel, Switzerland; 2 Accelrys, http:\\accelerys.com

**Keywords:** Chemical registration system, Chemical database, Chemical cartridge, Molecule import

## Abstract

Small molecule chemistry is of central importance to a number of R&D companies in diverse areas such as the pharmaceutical, nutraceutical, food flavoring, and cosmeceutical industries. In order to store and manage thousands of chemical compounds in such an environment, we have built a state-of-the-art master chemical database with unique structure identifiers. Here, we present the concept and methodology we used to build the system that we call the Unique Compound Database (UCD). In the UCD, each molecule is registered only once (uniqueness), structures with alternative representations are entered in a uniform way (normalization), and the chemical structure drawings are recognizable to chemists and to a cartridge. In brief, structural molecules are entered as neutral entities which can be associated with a salt. The salts are listed in a dictionary and bound to the molecule with the appropriate stoichiometric coefficient in an entity called “substance”. The substances are associated with batches. Once a molecule is registered, some properties (e.g., ADMET prediction, IUPAC name, chemical properties) are calculated automatically. The UCD has both automated and manual data controls. Moreover, the UCD concept enables the management of user errors in the structure entry by reassigning or archiving the batches. It also allows updating of the records to include newly discovered properties of individual structures. As our research spans a wide variety of scientific fields, the database enables registration of mixtures of compounds, enantiomers, tautomers, and compounds with unknown stereochemistries.

## Background

### General introduction

Small molecule chemistry is of central importance to a number of R&D companies in diverse areas, such as the pharmaceutical, nutraceutical, food flavoring, and cosmeceutical industries. These institutions all face similar problems, such as how to register and store information regarding small molecules in their corporate collections. The registration of compounds becomes even more complicated when two or more compounds have to be registered together as a mixture that has a particular mixture-specific property. Generally, people working on such projects have to find answers to the same questions, namely, which technology to use, what type of data need to be stored, how to manage physical samples of molecules, how to define the uniqueness of chemical structures, and how to make sure that the chemical structures entered by the chemists are drawn correctly. Surprisingly, this topic is rarely covered by scientific publications, and few insights can be gained from chemoinformatics books [1-5]. This is partly because it is a very technical challenge that is met by developers of the registration systems and partly because it is a rapidly evolving field.

In this paper we show how a chemical registration system can be built that we call the Unique Compound Database (UCD) and implemented at the corporate level of companies working with chemicals. Some elements of this system have been presented at two congresses [6-8]. Here, we provide examples from our experience of building a chemical registration system at Philip Morris International, Inc. (PMI).

**Figure 1:**
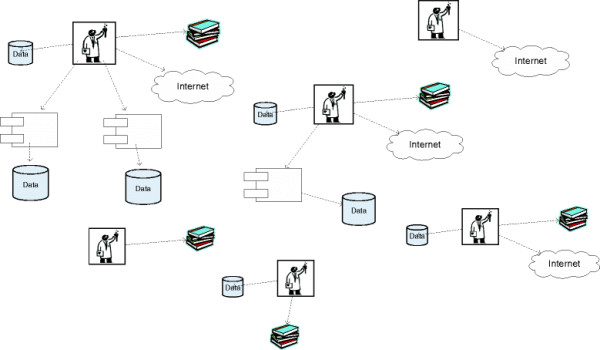
The challenge of connecting different data formats, activities, and scientific approaches without a designated data registry.

Without a common chemistry registration platform, chemical information is generally retained in a variety of locations, as illustrated in Figure 1. Lists are kept at a team or even at a scientist level in diverse formats, such as Excel files or Isis Base [9]. Transferring the data from several locations to a single registration system poses a challenge, because much of the information related to the molecule’s prior registration is incomplete (e.g., molecular data, scientists working with the molecule, name of the project) and there is often no molecular structure available for a molecule, just the name.

### Quality of chemistry representation

There is a wide range of chemical cartridges available on the market using different underlying database technologies (see Table 1). Chemical cartridges are basically database plug-ins that give chemical handling functionalities to the database. Cartridges usually differ in their performance, searching mechanism (exact match, substructure, similarity, etc.), and the ability to store large quantity of structures in the most compact way. While these are critical elements, we believe that there is another element that is usually underestimated: the concordance of the input system used by the end user (sketcher) and the underlying cartridge. These usually share the same chemical representation library, but full alignment between them is not always certain. The UCD overcomes this problem by ensuring that molecules are drawn and stored in the exact same manner.

**Table 1:** Examples of chemical cartridges

**Name;*web source***	**Database type**	**Available for free**
Accelrys Direct; http://www.*accelrys.com/products/informatics*	Oracle	No
Accelrys Accord; http://www.*accelrys.com/products/informatics*	Oracle	No
CambridgeSoft Oracle Cartridge; http://www.cambridgesoft.com/solutions/ details/?fid=186	Oracle	No
ChemAxon JChem; http://www.*chemaxon.com/jchem/intro*	Oracle	No
IDBS Activity Base; http://www.*idbs.com/products-and-services/ activitybase-suite*	Oracle	No
GGA Software Services Bingo; http://ggasoftware.com/opensource/bingo	Oracle and SQL Server	Yes
MolSoft MolCart; http://www.*molsoft.com/molcart.html*	MySQL	No
MyChem; *http://mychem.sourceforge.net*	MySQL	Yes
Pgchem tigress; *http://pgfoundry.org/projects/pgchem*	PostgreSQL	Yes
Orchem; *http://orchem.sourceforge.net*	Oracle	Yes

### Challenge of registering diverse data

The source of chemical compounds to be stored in a chemical registration system depends greatly on the business of the company. For instance, in pharmaceutical companies, compounds are synthesized internally or purchased from external suppliers and can represent millions of individually identified chemical structures. In the flavor and fragrance industry, compounds are often natural products extracted from plants.

For the tobacco industry in general, and for PMI R&D in particular, chemical constituent sources are relatively limited in comparison with traditional pharmaceutical inventories. Approximately 8400 compounds have been identified from tobacco plants and tobacco smoke [10]. However, we made sure that our system is as universal as possible and can hold millions of compounds as well. The challenge posed by the implementation of a registration system in this context is not the number of compounds to be registered, but rather the wide range of different chemistries represented (peptides, natural products, sugars, and complex products resulting from tobacco combustion). As such, a critical step in the project was to identify what we need to register and how to ensure that the quality criteria were met effectively.

### Design of the UCD

Our intention was to build a registration system of the compounds we use, not to create an inventory; therefore, the physical location and quantity of the chemical compounds were not considered. A project team of three chemoinformaticians supported by a project manager was responsible for defining needs and user requirements. Most of the requirements were related to chemical structure representation and storage; the starting point was that it must be possible to create and modify structures in the platform and to record physical samples attached to their related compounds.

For the purpose of data uniformity and uniqueness, the chemoinformaticians specified that the structures entered in the system have to be standardized prior to registration, and that uniqueness should be defined at the level of neutral molecules. In consequence, when a new batch is submitted, if a duplicate of the corresponding compound is found in the database, the system must create a new batch for the compound. It is also important for users to have the possibility to register compounds for which structures are not known. Such cases are annotated as “No Structure”, meaning that no particular chemical structure is defined. This is useful, for example, for analytical chemists working with mass spectrometry who might encounter the same peak in several gas chromatography and liquid chromatography mass spectra, without being able to identify the compound. For salts, the neutral form of the molecule is drawn and associated with the appropriate counter ions and ratio. In the same manner, hydrates are not drawn, but are associated with the chemical structure. The system must, therefore, be able to verify the uniqueness of the molecule regardless of whether it is in the form of a salt or a hydrate. The canonical representation is generated for each structure by the system, and the different tautomers of the same molecule must have the same canonical representation.

A temporary *staging* area (submission area) was planned to enable the storage of new molecule entries until they are validated by an expert before final registration in the system. In addition, three options were created to search by chemical structure: exact search, substructure search, and similarity search.

It was also important to define how batches should be managed by the person in charge of validating the data entered in the system. Batch reassignment was defined as an important feature of the system; batches can be reassigned from one molecule to another molecule by the registrar. This functionality also had to be available for the batch assigned to a molecule with « no structure«, for when the structure of the molecule is finally identified. To ensure that no information is lost during the process, an audit trail of the modification must be kept. In the same way, it should be possible for the person in charge of the system to archive batches, but not to delete them.

Technical requirements to make the system compatible with our technical infrastructure were also defined. For example the system should be compatible with Oracle 11 g [11], Windows Server 2003 [12], VMware ESXi [13] and Citrix [14]. In terms of performance, the system should be able to support 300 users through a centralized architecture, but five queries at the same time were considered the maximum. It was also imperative that data be available to external tools, and for this purpose a direct database access was requested in order to implement Extract Transform Load (ETL) processes [15].

The result of this process was a document containing 147 user requirements describing the platform.

## Concept of the UCD

### Data organization: Concept of molecule, substance, and batch

The three main entities in the UCD are molecule, substance, and batch. Definitions of these terms can be slightly different from one chemical system to another. In the UCD, the definitions are the following:

· *Molecule*: a neutral form of the chemical structure without any charge, counter-ion, or hydrate. If a molecule is charged, the system changes it to its neutral equivalent and records its salt form at the *substance* level. An exception was made for substances containing quaternary ammonium cations. The particularity of the quaternary ammonium cations is that they are permanently charged, independent of the pH of their solution. In this case, the system does not neutralize the molecule.

· *Substance*: a molecule (neutral or charged) with its counter-ion or hydrate.

· *Batch*: an occurrence of the compound in the company, generally a physical sample, identified from mass spectrometry, or a reference in the relevant literature.

**Figure 2:**
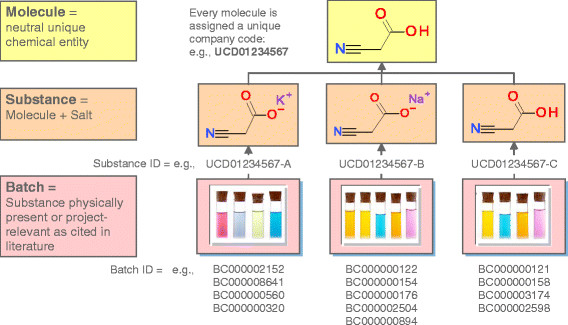
**Hierarchy of molecule, substance, and batch entities in the UCD. Example of the hierarchy is shown for a 2-cyanoacetic acid.** Three substances are associated with the molecule: two salts and one neutral substance. Each substance gets a coded letter after the molecule code (e.g., A in UCD01234567-A). Several batches are registered for each substance. Batch codes are generated incrementally upon registration and also serve as submission ID. Batches can be re-assigned to other molecules.

In the UCD, one molecule is linked to several substances and one substance can have several batches (see Figure 2). This allows users from different teams to enter batches (e.g., different laboratory procedures) of the same substance of the same molecule. This molecule can be a new one or one already registered in the UCD. One batch can contain only one substance, which in turn can contain only one molecule; however, one molecule can be represented by several different salts (substances) and each substance can have several batch entries. An example is presented in Figure 2. Batches are stored with experimentally relevant information entered manually by submitters. At the molecule level, a single molecule is represented by a unique, property-independent code generated by the registration system. The substance codes are the same as the molecule code, but a distinct letter is added for each distinct salt. Batches have their own unique codes. Batch codes are generated incrementally upon the submission process of the registration. Batch codes also serve as the Submission ID to submitters and registrars.

Each level requires specific information that is either entered manually by the scientists during the submission step of the registration or calculated automatically by the system. For example, the information related to the project, scientist, laboratory notebook reference, and analytical results is stored at the batch level (manual entry). Information related to the chemical substance, i.e., IUPAC name, codes (InChI and SMILES), and physicochemical properties such as molecular weight and logP, is stored at the substance level. Analogically, the same kind of information is stored at the molecule level for the neutral chemical structure.

The IUPAC names, SMILES, and InChI codes generated by the UCD can be canonical and/or can only partly represent the stereochemistry. When a molecule contains more than one group of relative stereocenters the chemical line notations SMILES and InChI are not sufficient to correctly represent the stereochemistry. For example, the ‘either’ bond (wavy line) pointed to a stereocenter cannot be encoded in InChI or SMILES codes. To represent the stereochemistry as precisely as possible we used the Symyx enhanced stereochemistry labeling (see section *Use of enhanced stereochemistry*). For such cases registered in the UCD, it is the detailed 2D drawing of the structure with enhanced labeling that assures the uniqueness of the molecule. In order to omit the ambiguity and be sure we are working with a single unique structure, the UCD generates a unique UCD code.

In the UCD, SMILES and InChI are still generated because these popular chemical line notations are useful to do some queries in external databases and the unique UCD code is an internal company code. However, it should be noted that considerable progress to guarantee uniqueness of the structural description using line notations has been made, as it can be seen in two recent publications [16,17], introducing and discussing yaInChI and CTISMILES codes, respectively.

In addition, the UCD allows the registration of mixtures of enantiomers. Because mixtures of the same enantiomers in different ratios (e.g., 50:50 and 30:70) may have different physico-chemical properties, the UCD generates a unique code for each ratio. Importantly, even if different enantiomers or mixture of enantiomers of the same molecule have different UCD codes, a search using the structure of the molecule will retrieve all the entries.

### Dealing with chemical structure

#### Chemical cartridges

The core technical functionality of the registration system is to handle chemical structures. Basically, this means that each chemical structure must be stored as a unique representation, and that the drawing of the structure must include all structural information, such as stereochemistry, so that users have the possibility to search by exact structure, substructure, or similarity.

Although it is possible to generate unique codes and do searches using classical chemoinformatics tools such as Pipeline Pilot [9] or Chemistry Development Kit [18], we believe the most suitable approach is to use a chemical cartridge as the central core of the registration system. Chemical cartridges have the advantage of offering very good performance (for the search of compound), because chemical structures are indexed in the database. In order to match our design and concept of the UCD, we chose the Accelrys Direct (formerly Symyx Direct) chemical cartridge (see in Table 1).

#### Use of enhanced stereochemistry

One of the main difficulties with chemical registration systems is the representation of uncertainties of stereoconfigurations and mixtures of stereoisomers. A common challenge in chemical structure registration is to represent stereogenic centers precisely, even when the absolute configuration is not known. We solved this issue by using a leading system of stereo centers description, i.e., Accelrys enhanced stereochemical representation (V3000 format), which uses embedded labels in the structure to allow precise configuration of the molecule for each possibility.

**Figure 3:**
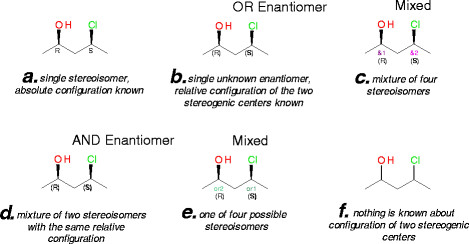
Different cases of stereochemistry managed by Accelrys Draw and Accelrys Direct chemical cartridge.

For example, the configuration of the two centers of 4-chloropentan-2-ol in the mixture of stereoisomers can be known or partially known. Embedded stereochemical labeling allows us to represent the relative stereoconfiguration of stereogenic centers. As seen in Figure 3, the six stereoisomers and stereoisomeric mixtures would be registered as six different molecules in the UCD; thus, removing the uncertainty of known or unknown stereoconfiguration.

#### Rules for drawing molecules

**Figure 4:**
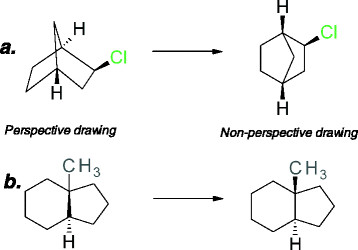
**Representation of molecule.** To be correctly understood by the chemical cartridge the drawing must be non-perspective (***a***), and the stereo bond in ring must be avoided (***b***).

**Figure 5:**
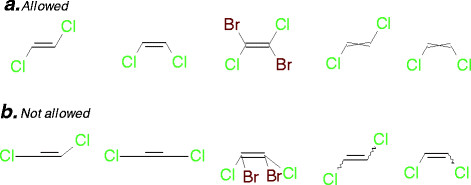
**This figure represents the allowed (*a*) and not allowed (*b*) drawing for the isomerism cis-trans.**

Because some molecules can be drawn in more than one way, it is important to define chemical drawing rules that disallow some representations, thus ensuring seamless translation of structure from the chemist to the chemical cartridge. In order to be correctly understood by the chemical cartridge, the structure representation must be done in a non-perspective way (see ***a*** in Figure 4). When a structure contains bridging atoms, the bonds that are attached to the bridging atoms should not be marked as stereo bonds; instead, the explicit hydrogens should be used (see ***b*** in Figure 4). The drawing of the cis-trans isomerism is also important. Figure 5 shows some examples of the type of drawings that are allowed (***a***) and not allowed (***b***).

**Figure 6:**
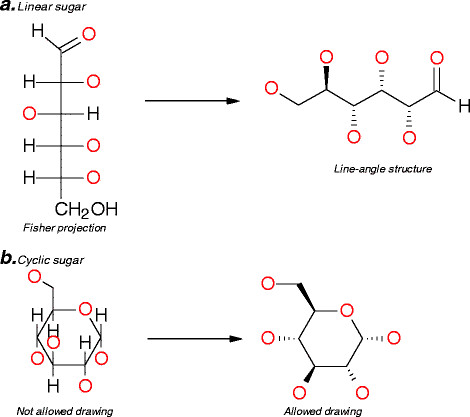
**Allowed representation of sugars.** This figure represents the allowed drawing for open-chain form of sugar of α- d-Glucose (***a***) and of cyclic form of d-Glucose with the allowed drawing based on the Haworth projection α-d-Glucose (***b***).

Finally, sugars can be represented in different ways, but only some of them are fully interpretable by the chemical cartridge. For linear sugars, the preferred drawing uses the line-angle structure rather than the Fischer projection [19] (see ***a*** in Figure 6). In a Fischer projection, the horizontal lines represent bonds coming out towards the observer and vertical lines represent bonds going away from the observer behind the plane of the paper. Cyclic structures of monosaccharides are represented using the Haworth projection [20] with a non-perspective drawing (see ***b*** in Figure 6). Haworth projections are easy to translate into acceptable drawings. Bonds above the plane of the carbon ring are marked “Up”, and bonds beneath the plane of the carbon ring are marked “Down”. Hydrogen atoms, which are explicit in Haworth projections, are implicit in structures that are drawn for registration.

#### Standardization and validation of compound representation

**Figure 7:**
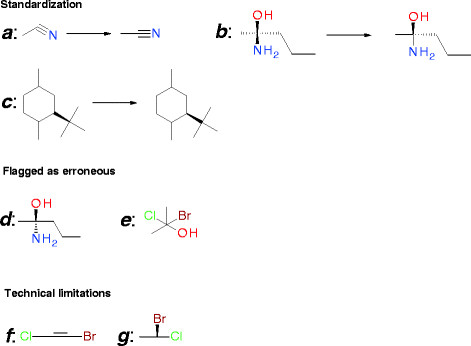
**Examples of standardization rules and error check.** (***a-c***) Some standardization rules are applied automatically by Accelrys Cheshire. (***a***) Nitrile group can be automatically redrawn linearly. (***b***) For a stereocenter it is not necessary to have two double up bonds and/or two double down bonds. (***c***) Up and/or Down bonds must be oriented to the stereocenter. (***d-e***) Some compounds can not be corrected, but can be detected as erroneous*.* (***d***) in this case it is not possible to determine the stereochemistry. (***e***) Valence is not correct. In some cases it is not possible to correct or detect automatically the error. (***f-g***) Some drawings meet technical limitations. (***f***) The configuration of the stereobond can not be determined. (***g***) The configuration of the stereocenter can not be determined.

Drawing rules provide the necessary help for scientists to represent chemical structures in a manner which is understood correctly by the chemical cartridge. However, because all structures must be checked automatically before they are entered in the database, some standardization rules must be automatically applied. We chose for this purpose Accelrys Cheshire (formerly Symyx Cheshire [9]), a chemistry-oriented scripting platform. With this tool it is possible to apply corporate standards to check and adjust chemical structures and neutralized structure. Some examples of standardization rules and error checks are presented in Figure 7.

**Figure 8:**
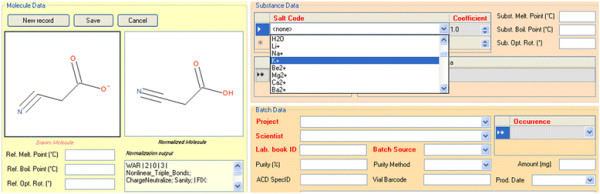
**Example of a standardization of the structure in the interface of the registration system of our company.** The nitrile group is drawn linearly and the acid group is put in the neutral form (Molecule Data). The output of the normalization script (Normalized Molecule) is presented to the user, who can accept the changes prior the submission of the molecule. Salt can be selected at the Substance Data level.

In some cases, structures can be standardized or checked automatically. In addition to an automatic check of the structure drawing by Accelrys Cheshire, the good drawing rules and validation by an expert established in the company help ensure that only correct structures are stored in the database. An example of the validation of chemical structures with Accelrys Cheshire in our platform is presented in Figure 8.

### Registration workflow

**Figure 9:**
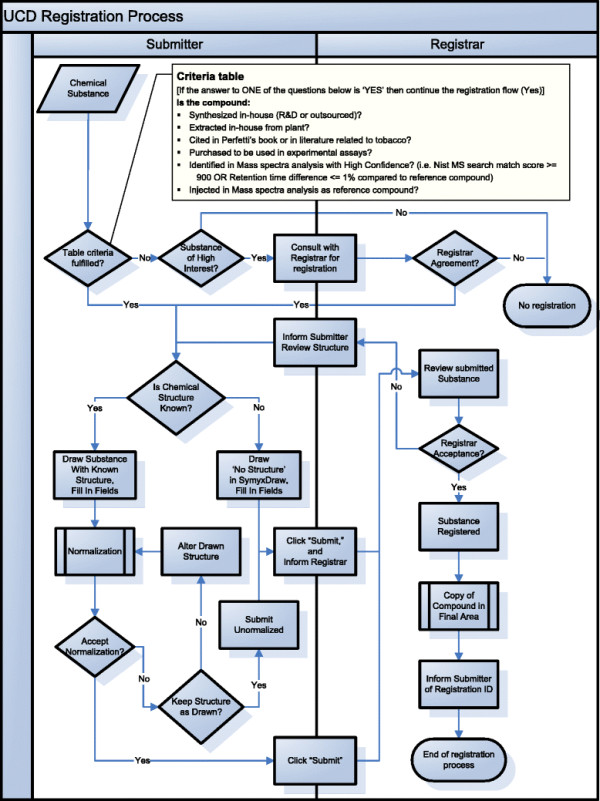
**UCD Registration workflow.** Tasks to be followed by Submitters (left part) and Registrars (right part). Criteria table defines which compound is to be registered.

When submitting a molecule to be registered in the UCD system, the submitters have to follow a registration workflow (Figure 9). Chemists wanting to add a new batch to the UCD should consider whether the chemical substance fulfills the criteria for inclusion in the database (see criteria table in Figure 9). If the chemical structure should be in the UCD, chemists must complete a form with the drawing structure. In cases where the exact structure of the compound is not known, the user can use the “No Structure” function of Symyx draw to fill in the form. This form also contains various fields for data associated to the batch (e.g., common name, source, internal identifier). When the user validates the drawing of the molecular structure, a normalization process is automatically executed, and the normalized structure is also displayed in the form (see example in Figure 8). The user must then decide whether or not to approve this normalization. When the form is complete, the user must click the Submit button to copy all the data into a transitory area that we call submission area. This area contains the compounds that have been submitted by users, but not yet validated. An automated control ensures that a molecule can be submitted only if all mandatory data and all the validation fields are passed (e.g., experimental molecular weight field accept only number). Once a molecule is submitted, there is a manual review and approval by a chemoinformatician. Approved molecules (and all related information) are copied into the registration area, which is the final database. When a submitted molecule is not approved, the chemist who submitted this molecule has the option to modify and resubmit it. The user roles for this workflow registration are described in the next section (*Roles*). This two-stage quality control process ensures the accuracy of the data.

In addition, once a molecule is in the registration area, we can manage the potential user errors in the structure entry by reassigning or archiving the batches. The system also allows the records to be updated to include newly discovered properties of individual structures. This batch reassignment process will be explained in section *Batch reassignment*.

### Data validation

Validation of data registered in the chemical registration system is imperative to ensure the reliability of the content. In our case, the two staging areas allow scientists to be part of the registration process and the registrar to check discrepancies and errors. However, because we decided that not all scientists should be able to create new records, we defined three roles.

#### Roles

**Figure 10:**
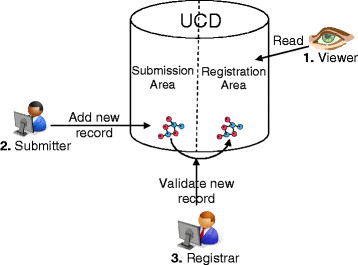
Three user roles and two staging areas of the database.

Authentication to access the database is managed by Lightweight Directory Access Protocol (LDAP) accounts with three groups corresponding to the three roles. Depending on the group that the user belongs to, different permissions can be assigned (Figure 10).

· The role of *Viewer* includes all R&D users that have access to the UCD. Viewers can search and view registered data via a web interface (except for some “restricted fields” reserved for specific teams).

· The role of *Submitter* is reserved for scientists who create new information. Submitters have the same privileges as Viewers, but can also access to a submission form to insert new records in the database.

· The role of *Registrar* is restricted to chemoinformaticians. Registrars have the same privileges as Submitters, but are also responsible for checking and validating data in the submission area (source, project ID, etc.) and giving approval for registering an entry.

As each user also belongs to a team group, when data associated with a molecule from a team are business sensitive, the UCD allows the information to be entered in a specific restricted field (e.g., study activity) that can be viewed by this team only.

#### Batch reassignment

A batch can be reassigned to a different substance if the user realizes that the structure was entered incorrectly or determines stereogenic centers during later experiments. If a substance no longer has a batch, the substance is archived as inactive. If after this process a molecule no longer has a substance, the molecule is archived. There is no ‘delete’ process; all new entries are assigned chronologically with new codes. This procedure allows for correction of errors without losing any information related to the archiving process.

#### Automatic calculations

In a chemical database, it is suitable to have names and certain properties (such as ADMET) calculated automatically. Even though our system generates corporate compound IDs (UCD codes) upon the registration of each entry, it is important that chemical names and other identifiers, such as IUPAC names or CAS numbers, be recorded to provide links to molecules in external databases. ACD/Labs Name Batch tool is used to automatically and accurately generate most names according to guidelines of the IUPAC from the molecular structure. However, naming structures with enhanced stereochemistry is an issue: see example of (2R,4 S)-4-chloropentan-2-ol in Figure 3 for which IUPAC names with such detailed description of stereochemistry cannot be generated. The software Accelrys Pipeline Pilot is used to predict ADMET properties and to calculate lead-like and Lipinski indicators. ACD/Labs PhysChem is used to automatically calculate water solubility values for the molecules.

**Figure 11:**
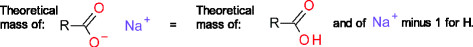
**The process of calculation of the final theoretical mass of the substances.** Normally, the molecule form is hydrogenated by the system, but the final mass is calculated correctly by the sum of the values of the acid and counter ion minus one hydrogen.

In addition, the system automatically calculates the theoretical molecular mass of both molecule and substance. The system neutralizes the charge atom of the molecule by adding one hydrogen to this molecule and the submitter selects the corresponding counter ion from the predefined dictionary of salts (for an example, salt of the carboxylic acid in Figure 11) for substance. As the charged form of the molecule is not represented in the database, the molecular mass of the salt is decreased by the theoretical molecular mass of one hydrogen to automatically calculate the theoretical molecular mass of the substance (Figure 11).

### Implementation of the platform

#### Implementation approach

It is known that the implementation of chemical registration systems can be a lengthy and difficult process. It was critical from the start that the project be clearly delivered in the shortest timeframe possible and that the implementation team be built to ensure maximum efficiency. As mentioned previously, a team was put together of three chemoinformaticians, one project manager, and the support from the former Symyx consulting team. This team was empowered by management to make all decisions regarding the chemical representation and standardization rules, the data structure, and the technical implementation strategy. A production system was available four months after the initial kick-off meeting.

#### Database

**Figure 12:**
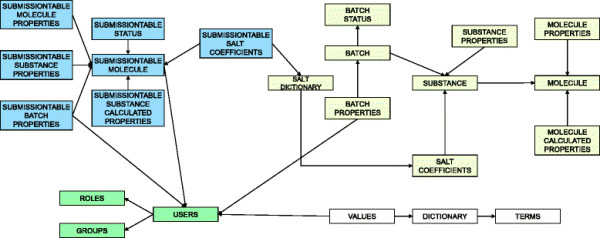
**Database model of Unique Compound Database.** Database is composed from tables related to submission area (blue), registration area (yellow), security (green) and additional properties (white).

The database is hosted on Oracle 11g with Symyx Direct 6.3 cartridge. The major effort in the design phase of the project was to conceive the database model (Figure 12). We saw that data are inserted in the database in two steps (submission and registration area). These two areas are clearly separated in the data model. The submission part of the scheme is a buffer area for the data containing all the information entered by the user. The registration tables contain the validated information. Molecule, substance, and batch tables reflect the organization of the chemical data presented before. Properties are stored in dedicated tables for each level of information. The information related to security and additional properties is stored in separate tables (green and white in Figure 12).

#### Software

**Figure 13:**
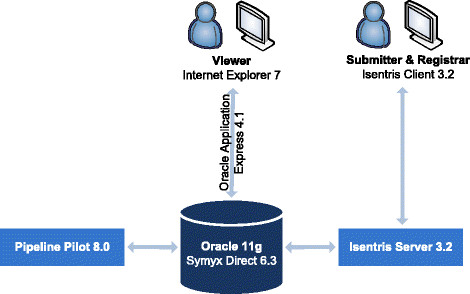
**Software architecture of the UCD.** The database part is based on Oracle and Accelrys Direct (formerly Symyx Direct). The software to input data is a desktop application based on the Isentris platform. The visualization interface was developed using Oracle Application Express and is accessible with a web browser. Pipeline Pilot is used as an ETL to compute physicochemical properties and chemical names overnight.

Several programs were used to build the UCD platform. As previously mentioned, data are stored in an Oracle 11g database using Accelrys Symyx Direct 6.3. Pipeline Pilot 8.0 is used to compute overnight for any new molecule entry the physicochemical properties (e.g., molecular weight, logP) and IUPAC names (generated by ACD/Labs Name). Symyx module Isentris was used to develop the main part of the platform (Figure 13). The Isentris platform offers a visual editor to build data sources on the top of Oracle in order to access the data without SQL code. Two data sources were built for the project, one for the submitter and one for the registrar. Then a desktop application was built using Isentris form designer. The application is executed using Isentris Client. It is used by submitters to enter new molecules and by the registrar to validate the data.

The number of submitters is limited in our company, but many people were interested to consult the data. We wanted a more flexible solution for the people who are only interested in viewing the data. We then chose to develop a web interface using Oracle Application Express 4.1 (Apex) to visualize the data in the UCD. Apex is a tool integrated by default in Oracle 11 g and dedicated to build web interfaces for Oracle databases in a very efficient way. The web interface is available to a large number of users in our company.

#### Data migration

Once the UCD was ready, a critical step was the migration of all the existing data. As is often the case, our scientists had the names of the molecules and some information about them stored locally in diverse file formats such as Excel or text (e.g., CAS number, experimental molecular weight, origin of the compound). The following section describes the challenges involved in transforming names into structures and enabling automatic import of molecules into the UCD.

##### Transformation of names into structures

In building the UCD without any existing infrastructure, we had two major sources of compounds available: 1) names of molecules as listed in the literature and 2) internal working compounds, which are often referred to by their IUPAC names, common names (e.g., harmane), or CAS numbers.

**Figure 14:**
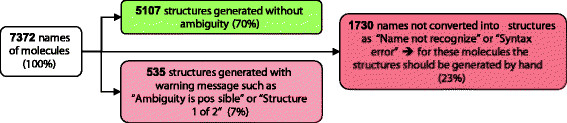
**Success rates for converting chemical names to structure.** In this example around 70% of the names were transformed correctly into structures, 7% structures of the generated structures were ambiguous, and 23% of the names were not recognized by the software.

The names do not always follow the IUPAC recommendations; therefore, transforming the names into structures and importing them into the UCD was challenging. The issue was addressed using the standard software module ‘ACD/Name to Structure Batch’ from ACD/Labs [21]. This software generates accurate structures for entire libraries of compound names. As illustrated by numbers in Figure 14, even if this software can transform a large number of names into structures, a considerable amount of time must be spent by the submitters and registrars correcting the structural representation of molecules.

Of the 7372 molecule names entered, 70% of the structures were generated correctly; 7% of the structures were generated with warning messages, which required manual curation; and 23% of the structures were not generated because the names were not recognized by the software (Figure 14). For this subset of molecules there was no easy or automatic way to obtain a structure; therefore, the chemists had to check and correct each name and its structure manually. To obtain a structure for these molecules, we conducted searches in PubChem [22], ChemSpider [23], and Google [24]. For some molecules, an associated CAS number was available, which allowed us to obtain the structure using SciFinder® [25], a tool for exploring the CAS databases. Clearly, during the construction of our UCD, structure conversion took considerable time (in terms of months for one chemoinformatician) and the effort and time required should not be underestimated.

##### Automated migration workflow

**Figure 15:**
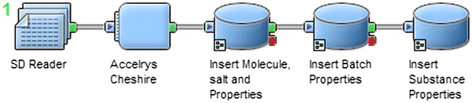
Accelrys Pipeline Pilot protocol to insert automatically an entire library of compound.

Once we have the structures, we should import all the compounds into the UCD. To avoid doing this import manually molecule by molecule, which would be extremely time-consuming, a Pipeline Pilot protocol was developed to automate the importation (Figure 15).

This protocol requires an input file in SDF (stands for structure-data file) format. In this input file the structure of the molecules is described by a Connection Table in V3000 format (as explained in section *Use of enhanced stereochemistry*). The SDF format was chosen because of its ability to include associated data. The input file contains information about the molecule structure and all the data associated with this molecule (e.g., Project name, internal identifier, scientist who works on this compound).

In order to ensure that structures are normalized according to the same rules as defined for the manual import, we developed a Java component for Pipeline Pilot which uses the Java API of Accelrys Cheshire to normalize chemical structures.

## Discussion and conclusion

At PMI R&D, we have built a chemical registration system called the Unique Compound Database (UCD), which manages the registration process in an efficient and non-redundant manner in a very short timeframe.

In order to register data efficiently and accurately, the UCD has the flexibility to register molecules with unknown structures or mixtures of compounds and at the same time can be used to register known structures with the precisely defined stereochemical configuration. This level of detail ensures the uniqueness of chemical records. Pre-defined standardization rules, drawing rules, automated normalization, and enhanced stereochemistry labeling decrease the chance of erroneous or ambiguous registry. Moreover, the system decreases the name-to-structure ambiguity by using only drawn structures (with the enhanced stereochemistry when known) and by generating names only after the registration process is completed.

The reliability of the database and the accuracy of the registration process are enhanced by the two-stage area. The *Submitter* or bench chemist takes ownership of the records and registers the records “*from the bench*”. This process is assisted by the automated standardization rules and automatic structure check. The *Registrar* reviews the submitted molecules and validates the structures before registration. The two-stage area system also allows the *Registrar* to detect potential software issues that the *Submitter* might have encountered.

Concerning the molecule-to-salt and salt-to-batch associations, our model prefers registering molecules as neutral entities, where predefined salts are listed in a dictionary and selected by the user at the substance level. Salts are standardized and do not have to be drawn. Batches are then assigned to the substance entry. We believe this process provides a higher level of molecule description and easier traceability of different entries. Furthermore, batches can be re-assigned or archived, thus providing the company a way to deal with new changes to the structures (i.e., structure elucidation) and to log such changes. We have also observed that actual data import into the newly built platform is the time-consuming and the most challenging step.

Finally, we believe that the UCD concept is an efficient and progressive way to accurately register and describe all structures at the corporate level. The database is modular and flexible. It allows us to link the accurately described molecules to other databases. Thus, uniqueness of molecular description in the UCD provides the robust foundation of the company chemical space. In consequence, compounds can be moved to complex knowledge bases and data can be mined for biological activities, modes of action, and therapeutic outcomes.

Future development of the UCD platform will include linking with the integration of spectroscopic information in relationship with the different entities stored in the system. We are also planning to move the entire system to a web-based architecture using the latest in sketcher technologies, which will increase the ability of the bench scientist to easily register any new substance. In addition, we will be integrating the chemical standardization rules within the database itself to simplify the maintenance process.

## Competing interests

The software that was used for the basis of the database development (from Accelrys) was selected using independent PMI review process. Subsequently, FG from Accelrys was employed to customize the development of the platform.

## Authors’ contributions

MCP conceived the project. JD and PP jointly designed the concept and managed the project. AM and FG did the development phase of the project. EM and AM provided input to the design, constructed the registration platform and they are the main authors for this manuscript. EM populated the database. All authors reviewed and approved the final manuscript.
